# Burkitt lymphoma associated with human immunodeficiency virus infection and pulmonary tuberculosis: A case report

**DOI:** 10.1097/MD.0000000000023853

**Published:** 2020-12-24

**Authors:** Victoria Birlutiu, Rares-Mircea Birlutiu, Ioan Sorin Zaharie, Mariana Sandu

**Affiliations:** a Faculty of Medicine Sibiu, Academic Emergency Hospital Sibiu, Romania - Infectious Diseases Clinic; b Faculty of Medicine, “FOISOR” Clinical Hospital of Orthopedics, Traumatology and Osteoarticular TB Bucharest, Lucian Blaga University of Sibiu; c Department of Pathology; d Department of Radiology and Medical Imaging, Academic Emergency Hospital Sibiu, Sibiu, Romania.

**Keywords:** Burkitt lymphoma, dose-adjusted etoposide, doxorubicin, and cyclophosphamide with vincristine, prednisone, and rituximab, highly active antiretroviral therapy, human immunodeficiency virus infection, pulmonary tuberculosis

## Abstract

**Introduction:**

The association of human immunodeficiency virus (HIV) infection with Burkitt lymphoma is related to the presence of Epstein Barr virus infection and the impact of the HIV antigen on the expansion of B-polyclonal cells. In Southeast Europe, the association is rare, and recognizing this is important in the therapeutic decision to increase patient survival rate. The association of HIV with Burkitt lymphoma and tuberculosis is even more rarely described in the literature.

**Patient concerns:**

We present the case of a 40-year-old patient who presented with a 3-week history of fever (max. 38.7 °C), painful axillary swelling on the right side, lumbar pain, gait disorders, headache, and night sweats. Clinical manifestations included marked weight loss (about 30 kg in the last 2 months before his admission).

**Diagnosis:**

A LyCD4 count of 38/μL and a HIV1 viral load of 384,000/mm^3^, classified the patient into a C3 stage. A biopsy of the right axillary lymph node was performed for suspected ganglionic tuberculosis due to immunodeficiency. Histopathological examination confirmed the diagnosis of Burkitt lymphoma. Cultures on Löwenstein-Jensen medium from sputum harvested at first admission were positive for *Mycobacterium tuberculosis*.

**Interventions:**

Highly active antiretroviral therapy, chemotherapeutic agents for Burkitt lymphoma, anti-tuberculous drug therapy, neurosurgical intervention of spinal cord decompression, and antibiotic therapy of the associated bacterial infection.

**Outcome:**

Burkitt lymphoma disseminated rapidly, with central nervous system, spinal cord, osteomuscular, adrenal, and spleen involvement. The evolution under treatment was unfavorable, with patient death occurring 6 months after diagnosis.

**Conclusions:**

The association of HIV infection with Burkitt lymphoma and tuberculosis is rare in the highly active antiretroviral therapy (HAART) era, posing prompt and multidisciplinary therapeutic management issues. Similar cases of HIV-TB and Burkitt lymphoma association have been described, but none of the other cases showed the involvement of the central nervous system or of the bilateral adrenal glands.

## 1 Introduction

The association of human immunodeficiency virus (HIV) infection with Burkitt lymphoma (BL) is 261 times higher than that in the general population and is linked to the presence of Epstein Barr virus infection and the impact of the HIV antigen on the expansion of B-polyclonal cells. In some areas, the incidence of BL in HIV patients is 23 per 100,000 persons (like in southern Louisiana), compared with the Southeast Europe where the association is rare.^[1]^ Recognizing this association is important for the therapeutic decision to increase patient survival rate. Concomitant administration of highly active antiretroviral therapy (HAART) with Burkitt lymphoma chemotherapy, dose-adjusted etoposide, doxorubicin, and cyclophosphamide with vincristine, prednisone, and rituximab (DA-EPOCH-R), is associated with good efficacy and tolerance, thus improving the prognosis of the disease. We present the case of a 40-year-old patient with HIV infection associated with disseminated Burkitt lymphoma, with central nervous system, spinal cord, osteomuscular, adrenal, and spleen involvement and pleuropulmonary tuberculosis, a case that posed particular treatment problems.

## 2 Case report

We present the case of a male Caucasian patient, aged 40 years, who presented with a 3 weeks history of chills, and fever (max. 38.7 °C), painful axillary swelling on the right side, lumbar pain, gait disorders, headache, and night sweats. Clinical manifestations occurred in the context of a marked weight loss (about 30 kg in the last 2 months before his admission).

He was examined by a general practitioner, and then by a neurologist who recommended laboratory tests (results that revealed a biological inflammatory syndrome) and lumbar spine magnetic resonance imaging (MRI) to be performed. The lumbar spine MRI findings were as follows: an extradural intracanal mass lesion posterior to the L4 and L5 vertebrae possibly suggesting a hematic focal mass. Adjacent to the L4 spinous process, a paravertebral area with fluid densities suggesting an abscess or muscular contusion. Small edema like focal area anterior and inferior to T10 vertebra angle suggesting spondylodiscitis.

After neurosurgical assessment, the patient was admitted to the Infectious Diseases Clinic for further investigations due to suspicion of spondylodiscitis, persistence of the fever of unknown origin and the biologic inflammatory syndrome of unknown origin. At the time of admission, on physical examination, the following were noted: altered general condition, normal weight (body mass index [BMI] of 22.69 kg/m^2^), pallor, right axillary conglomerate of about 4 cm of lymph node masses, right mobile painless supraclavicular lymphadenopathy with a diameter of 1.5 cm, lower extremity myalgia, irritative cough, basal diminished vesicular sounds, interscapular bronchial rales, heart rate (HR) of 96 beats per minute (bpm), blood pressure (BP) of 120/80 mmHg, 2 cm hepatomegaly, walking abnormalities, and diminished tendon reflexes.

The main laboratory examinations that were performed are presented in Table 1.

**Table 1:** Evolution of the main laboratory examinations during hospitalization.

Parameter	Values						Reference value
	02.05.2018	11.05.2018	02.07.2018	13.07.2018	17.07.2018	19.07.2018	
Hemoglobin	11.3/dL	10.1 g/dL	10.5 g/dL	9.9 g/dL	9.4 g/dL	8.4 g/dL	12–15 g/dL
Hematocrit	33.0%	29.9%	30.7%	27.9%	26.4%	24.1%	37–47%
WBC	7.060 × 10^3^/μL	4.320 × 10^3^/μL	2.640 × 10^3^/μL	0.19 × 10^3^/μL	0.20 × 10^3^/μL	7.72 × 10^3^/μL	4–10 × 10^3^/μL
Differential blood count:							
Neutrophils	54.3%	48.6%	40.2%	26.2%	25%	86.8%	30–75%
Lymphocytes	22.5%	27.5	34.8	57.9%	50%	2.7%	25–35%
Monocytes	19.4%	22.5%	24.2%	5.3%	10%	10.1%	2–10%
Eosinophils	3.8%	1.4%	0.4%	5.3%	5%	0.3%	1–4%
Basophils	0.0%	0.0%	0.4%	5.3%	10%	0.1%	0–1%
Thrombocytes	228 × 10^3^/μL	218 × 10^3^/μL	206 × 10^3^/μL	80 × 10^3^/μL	64 × 10^3^/μL	247 × 10^3^/μL	150–400 × 10^3^/μL
Prothrombin time, s	12.6 s	12.1 s	13 s				9.9–12.3 s
INR	1.11	1.06	1				0.86–1.1
Hemoculture	Negative						
Fibrinogen	488.8 mg/dL	572.9 mg/dL		397.5 mg/dL			170–420 mg/dL
C-reactive protein	54.22	81.68		61.47			<6 mg/dL
ESR	100	75		26			0–20 mm/h
AST	33 U/L			24 U/L			5–34 U/L
ALT	31 U/L			44 U/L			<55 U/L
Hepatitis B surface antigen	Negative						
Hepatitis C antibodies							
	Negative						
HIV antibodies	Positive						

A native thoraco-abdominal multiple detector computed tomography (MDCT) scan with contiguous sections was performed which revealed right axillary lymphadenopathy with a diameter of 46.5/27.5 mm, left axillary infracentrimetric lymphadenopathies, right adrenal gland tumor of 35/29 mm, and mediastinal infracentimetric lymphadenopathies.

A LyCD4 count of 38/μL and a HIV1 viral load of 384,000 copies/mL confirmed the HIV infection, and also classified the patient in a C3 stage according to the CDC.

A biopsy of the right axillary lymph node was performed for the suspicion of ganglionic tuberculosis due to immunodeficiency. Histopathological examination confirmed Burkitt lymphoma diagnosis.

**Figure 1:**
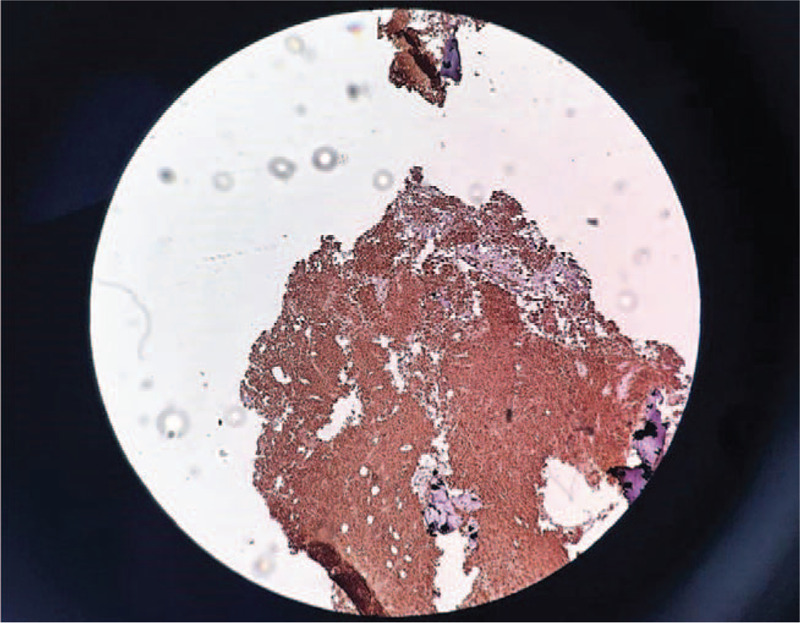
Histopathological aspect. Hematoxylin and eosin-stained sections of lymph node. Magnification ×100. Deletion of the follicular structure, diffuse proliferation with medium-sized atypical lymphoid cells, with large nuclei and prominent nucleoli, with numerous mitoses nuclei and with the presence of macrophage areas with intracytoplasmic detritus (“starry sky” appearance). Areas of necrosis of the “ghostly” aspect of the atypical cells.

Immunohistochemical staining of the lymph node revealed the following: few CD3+ and CD5+ reactive T lymphocytes; diffuse atypical positive lymphoid population for CD10 and CD20. On CD23 and CD30 staining, no positive lymphocytes were found in the atypical lymphoid population. BCL2 expression was negative on BCL2 immunohistochemical staining. In addition CyclinD1 and DBA44 staining were negative, with a Ki67 score of 90% (CD10+, CD20+, BCL2–, CycinD1–, Ki 67–90%, CD23–, CD30–) (Fig. 1).

For the same suspicion of ganglionic tuberculosis due to immunodeficiency, sputum was harvested for cultures on Löwenstein-Jensen medium. An acid-fast bacillus (AFB) stain for *Mycobacteria* on a sample of sputum was also performed. The stain was applied on a direct and concentrated smear. The result was a negative AFB smear.

Antiretroviral therapy with Raltegravir, Emtricitabine, and Tenofovir Disoproxil Fumarate was initiated. The recommendation was also made to initiate the Burkitt lymphoma treatment. Unfortunately, the patient seeking a second opinion postponed the beginning of the treatment.

The patient returned after a month to the emergency department with right facial paralysis, paraplegia, and loss of the sphincter reflex for micturition and defecation.

A computed tomography (CT) scan reassessment of the patient was performed, and revealed intracranial and intraorbital lesions with dural extension, invasion of the cranial bone structures, bilateral adrenal nodes (6 cm in diameter on the right side, and 4 cm on the left side), hepatosplenomegaly, bilateral pleural effusion, and multiple vertebral, sacral, and femoral lesions (complete description is available as supplementary material).

**Figure 2:**
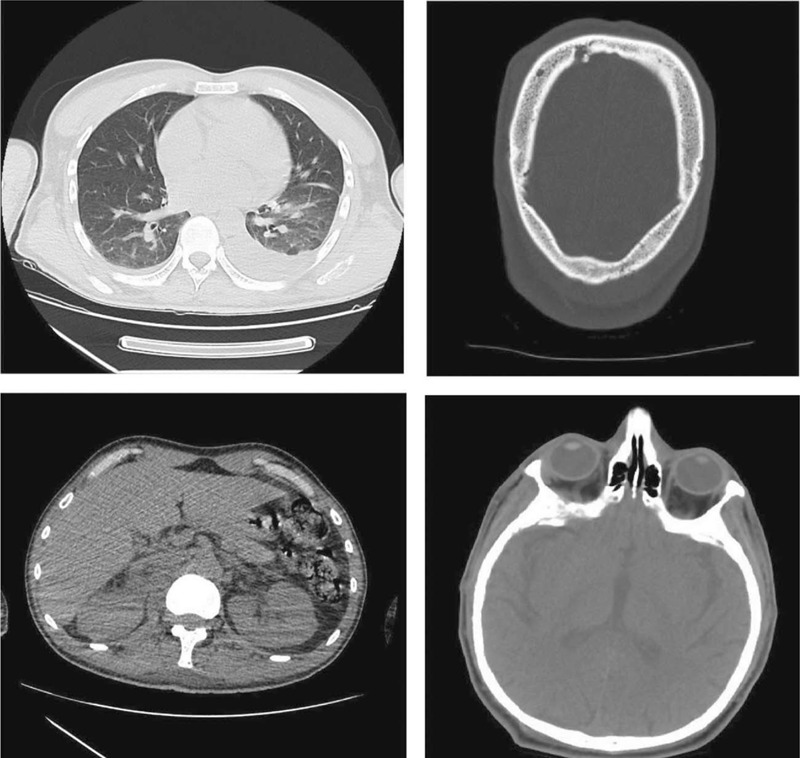
CT scan sections. CT = computed tomography.

**Figure 3:**
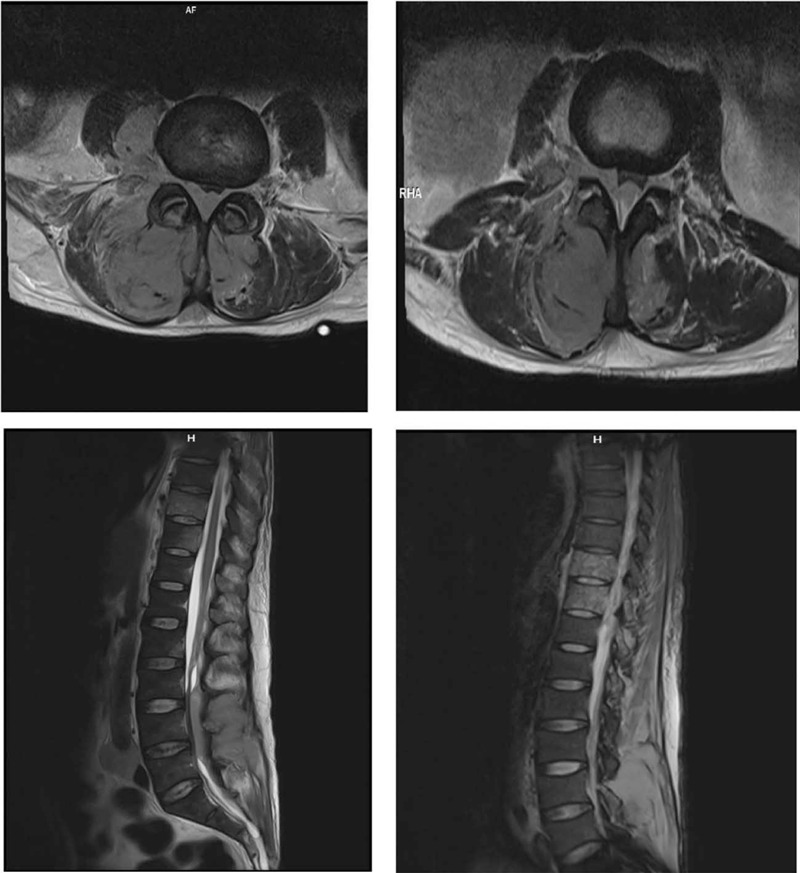
MRI sequence. MRI = magnetic resonance imaging.

**Figure 4:**
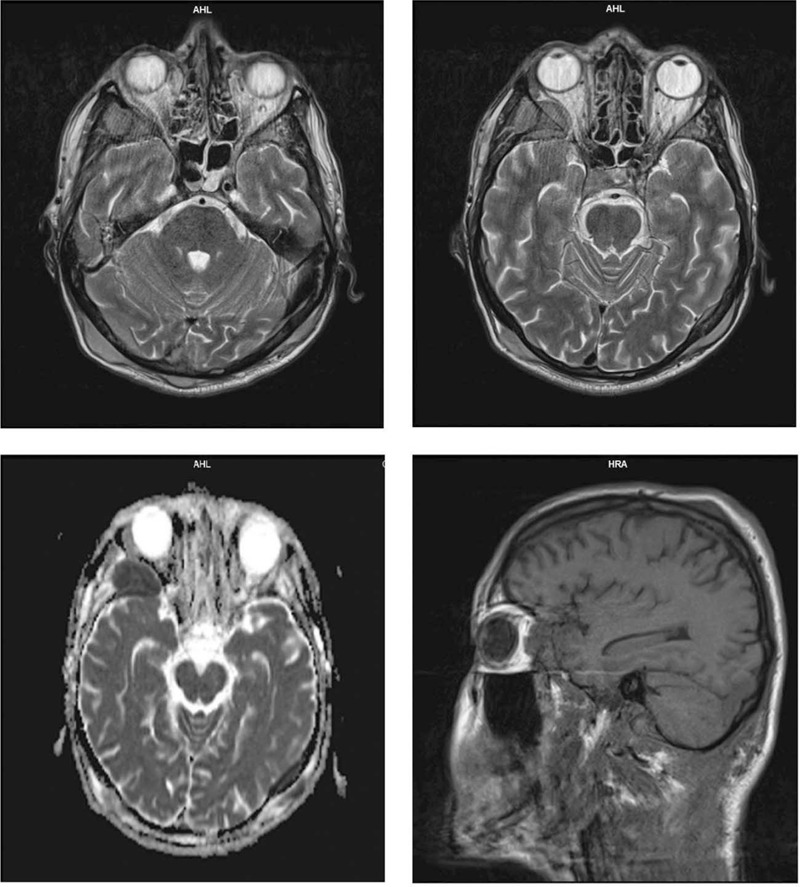
MRI sequences. MRI = magnetic resonance imaging.

Cranial and lumbar magnetic resonance imaging (MRI) was also performed and the findings were the following: multiple disseminated restrictive tumor masses in the scalp, with transosseous dissemination and dural intersection; bilateral restrictive, massive paravertebral muscle masses, larger on the right side, with intrartechal invasion from L2 to S2 and complete stenosis of the spinal canal at the L3 to L5 level. The presence of a prevertebral and para-aortic lymph node metastasis of approximately 3 cm was also confirmed (Figs. 2–4).

The patient was referred to the Department of Neurosurgery, where a L3–L4 and L4–L5 laminectomy was performed. Subsequently, he was referred to the Department of Hematology for the therapeutic management decision regarding Burkitt lymphoma. Biopsies from the excised tumors were taken, and histopathological examination confirmed Burkitt lymphoma. The microscopic appearance corroborated with the immunohistochemical aspect of positive lymphoid population for CD10, CD20, and KI67 and a negative expression of BCL2 indicates a Burkitt lymphoma, allowing its differentiation with diffuse large B-cell lymphoma and non-classifiable B-cell lymphoma with intermediate aspects between diffuse large B-cell lymphoma and Burkitt lymphoma.

Bone marrow examination revealed an infiltration by peroxidase-negative blasts cells of 6% to 36%.

DA-EPOCH-R therapy was initiated (rituximab 700mg-etoposide 96 mg day 1–4, sindovine 0.77 mg/d day 1–4, endoxan 1450 mg/d day 5, and doxorubicin 20 mg day 3–4) associated with filgrastim 48 MU SC on day 5, preceded by cyclophosphamide 200 mg, 2× 1 vial/d and prednisone 60 mg/m^2^/d for 5 days. The initiation of radiotherapy was delayed due to the medullary aplasia that occurred after the treatment, and it was initiated after the correction of the blood count.

**Figure 5:**
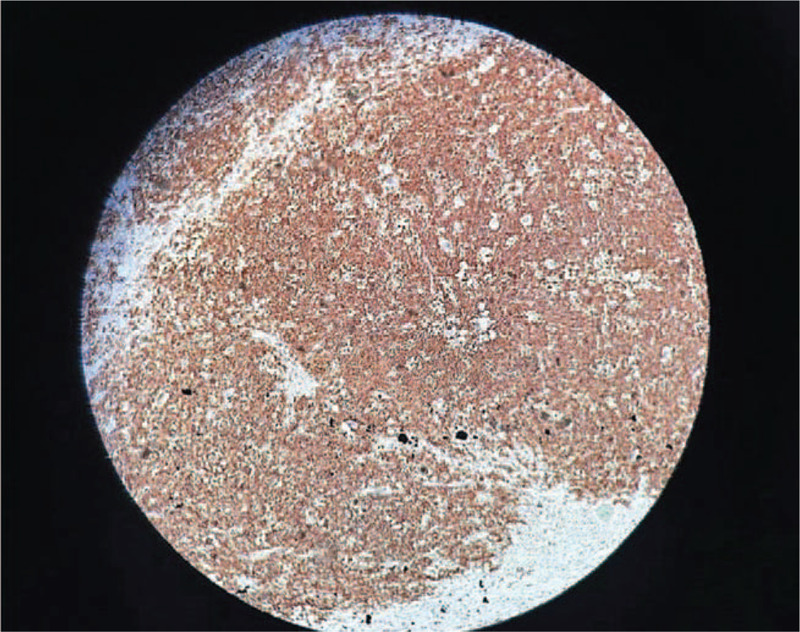
Histopathological aspect. Hematoxylin and eosin-stained sections of bone marrow. Magnification ×100. Partially preserved architecture with approximately 50% infiltration with medium-sized atypical lymphoid cells with large nuclei and prominent nucleoli, with extensive tumor necrosis. In less infiltrated areas, myeloid–erythroid ratio: within normal range. Hyperplastic erythroid population with less erythroblastic islets. Easily hyperplasic granulocytic population with slightly multiple precursors with lower maturation. Easily multiplied megakaryocytes in all maturation phases. Plasmocytes within normal limits.

**Figure 6:**
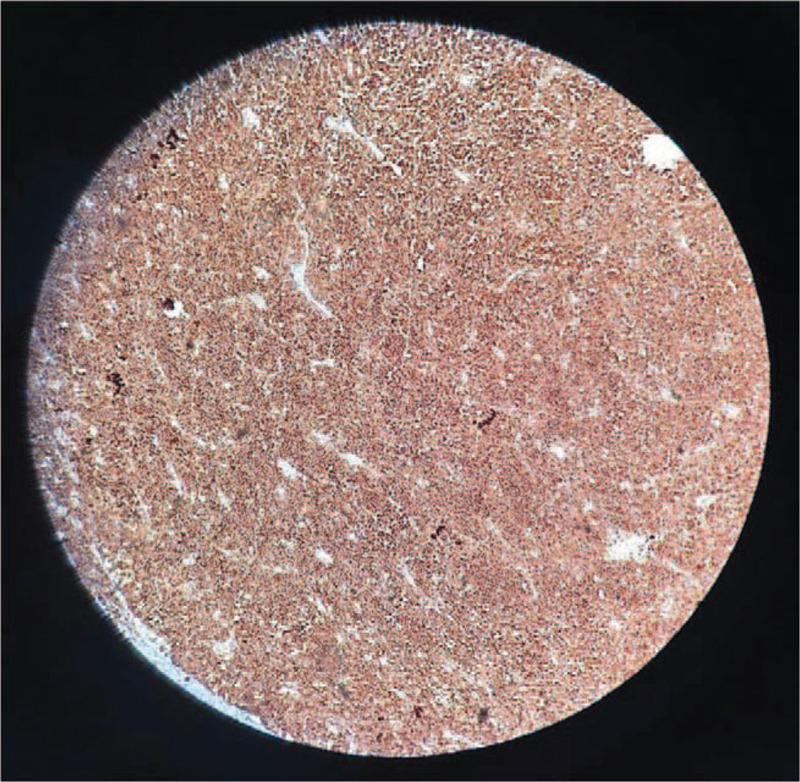
Histopathological aspect. Immunohistochemically staining of the bone marrow for CD10 and CD20. Magnification ×100. Positive in the lymphoid atypical population, except in the areas of tumor necrosis.

**Figure 7:**
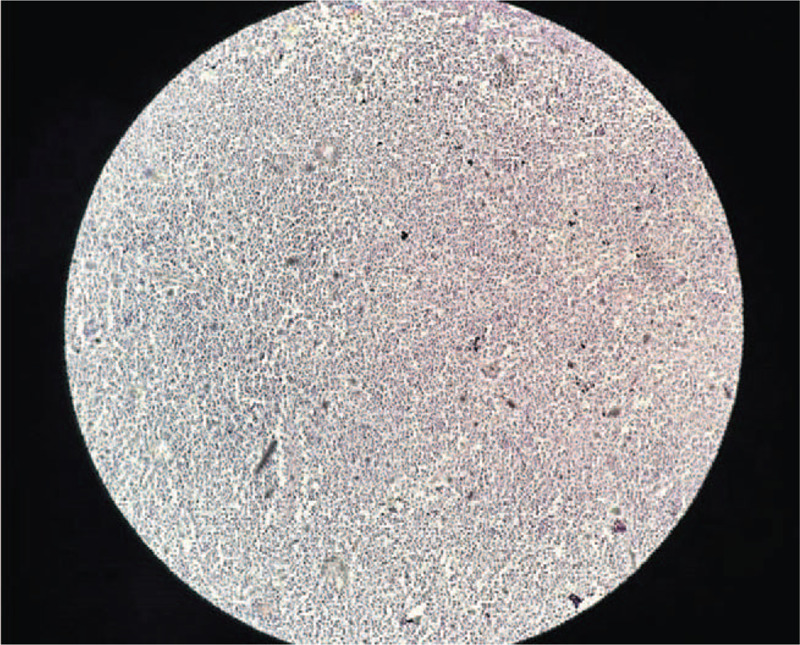
Histopathological aspect. Immunohistochemical staining of the bone marrow for BCL2 with negative expression. Magnification ×100.

**Figure 8:**
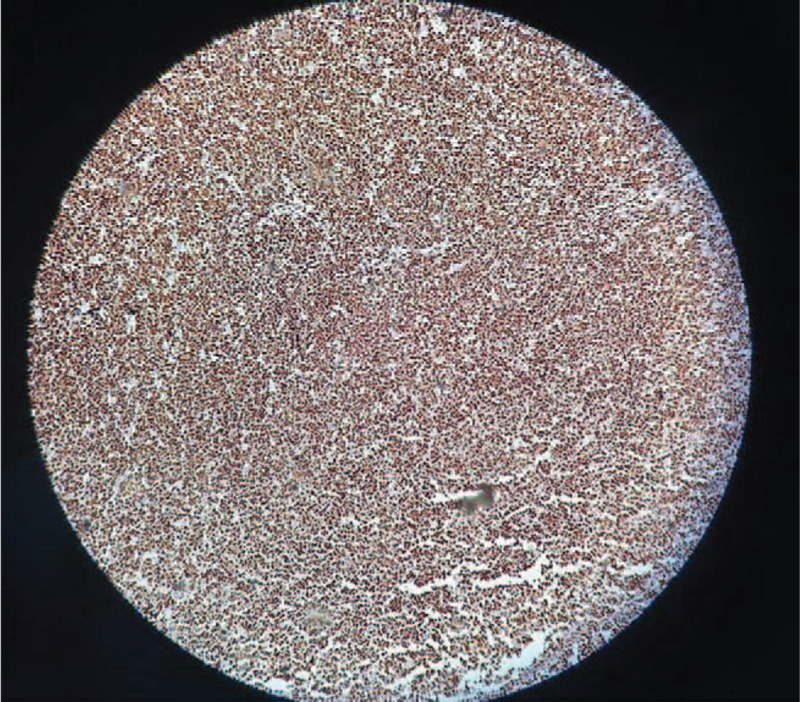
Histopathological aspect. Ki67 positive (80%) in the atypical viable lymphocyte population. Magnification ×100.

During the hospitalization period in the Department of Hematology, an episode of diarrhea occurred, which was confirmed to be related to a *Clostridium difficile* infection. *C difficile* infection was diagnosed according to the guidelines published by the European Society of Clinical Microbiology and Infectious Diseases using 2 highly sensitive tests: GDH EIA (glutamate dehydrogenase enzyme immunoassay) and toxin A/B EIA, both tests were positive. The episode of CDI has been managed with oral vancomycin 4× 125 mg per day for 14 days (Figs. 5–8).

Cultures on Löwenstein-Jensen medium from sputum harvested at first admission were positive for *Mycobacterium tuberculosis*, after 6 weeks of incubation.

Highly active antiretroviral therapy and Burkitt lymphoma chemotherapeutic agents were combined with anti-tuberculosis drug therapy (with rifabutin, pyrazinamide, ethambutol, and isoniazid). The evolution was unfavorable with the patient's death occurring 6 months after diagnosis.

## 3 Discussions

Burkitt lymphoma is the most aggressive form of non-Hodgkin lymphoma with B lymphocytes,^[2]^ with endemic or sporadic evolution, or being associated with immunodeficiency syndromes: HIV infection, congenital immunodeficiency syndromes or in allografted patients.^[3,4]^ It is characterized by a particularly high rate of proliferation (approximately 100%),^[5]^ with cells doubling time of 24 hours and cytogenetic specific changes of the c-MYC proto-oncogene. Cases associated with HIV infection represent 30% to 40% of non-Hodgkin lymphoma associated with HIV infection. Disseminated forms of BL with central nervous system involvement represent 20% to 30% of cases. In the history of HIV therapy, the association of HIV infection with non-Hodgkin lymphoma was 60 to 200 times higher than that in the general population.^[6]^

There have been cases of BL with lymph node, hepatic, and pancreatic localization,^[7]^ sometimes with an obstructive jaundice evolution.^[8–10]^ Also there have been cases of BL with cardiac involvement, concurrently with extradural lumbosacral lesions^[11]^ or ethmoidal sinuses and cerebral (temporal) impairment.^[12]^ Central nervous system involvement in HIV-infected patients is appropriate for differential diagnosis, especially in cases with focal neurologic deficits, with opportunistic infections such as cerebral toxoplasmosis.^[13]^ Disseminated BL with lymph nodes, central nervous system, and bilateral adrenal gland involvement associated with tuberculosis in an HIV-infected patient has yet not been described by other authors. The evolution of our case is extremely reserved, having as unfavorable prognostic factors, the CD4+ cell count below 100 cells/μL, age over 35 years, and an advanced stage of the disease (BL stage IV).^[14,15]^

Similar cases of HIV-TB and Burkitt lymphoma association have been described, cases with dissemination at an oropharyngeal, mediastinum, or pleural level,^[16]^ and none of these cases presented with involvement of the central nervous system or of the bilateral adrenal glands.

HAART therapy associated with DA-EPOCH ± Rituximab (administered according to CD4+ lymphocyte counts) appears to be associated with the best survival rate like CODOX-M/IVAC (cyclophosphamide, vincristine, doxorubicin, high dose methotrexate/ifosfamide, etoposide, and high-dose cytarabine).^[17]^

The present case describes an association of HIV infection with disseminated LB and pulmonary tuberculosis initially with the absence of imaging changes. Regarding the HIV-infected patient who is in the last stage of HIV infection (AIDS), the risk of reactivation of a latent TB infection increases by approximately 20-folds,^[18]^ and each third death is attributed to TB.^[19]^ According to the Joint United Nations Programme on HIV/AIDS, in 2016, worldwide, 1.2 million new cases of TB associated with HIV infection were recorded. According to the data published by the European Centre for Disease Prevention and Control, 58.994 cases were recorded in Europe in 2016, of which 13.617 cases in Romania,^[20]^ of which 9.222 cases with pulmonary localization, 10.5% being multidrug-resistant, and 5.6% pandrug-resistant TB forms,^[21]^ motivating the clinician's prudence in the investigation of TB in HIV-infected patients. Imaging changes may be absent in coinfected HIV/TB patients or may be atypical,^[21]^ so the decision to investigate patients for TB by smear, culture, Xpert MTB/RIF assay, or molecular diagnosis is absolutely necessary for countries where TB infection is endemic/epidemic.

The presence of mediastinal adenopathy may be the only imaging change in pulmonary TB in HIV-infected patients with CD4+ cell counts below 200 cells/μL.^[22,23]^ It is estimated that 60% to 68.8% of TB cases in patients in the last stage of HIV infection are associated with mediastinal/hilar adenopathy.^[24,25]^

In our case, the aspect of the CT scan of the thorax and a negative AFB smear seemed to be sufficient to rule out a possible HIV-TB coinfection, but the dynamic imaging changes correlated with positive cultures on Löwenstein-Jensen medium confirmed the diagnosis. Susceptibility testing of the *M tuberculosis* isolated strain demonstrated conservation of sensitivity to treatment and allowed the use of first-line therapy drugs (rifabutin, pyrazinamide, ethambutol, and isoniazid). TB treatment was initiated after the first month of HAART and Burkitt lymphoma chemotherapy, without developing immune reconstitution inflammatory syndrome. The patient partially recovered neurologically and was able to move his legs but only in a supine position. At 3 months after the initiation of HAART therapy the CD4+ cell count was 178 cells/μL. Unfortunately at 6 months the patient's death was attributed to Burkitt lymphoma.

## 4 Conclusions

Burkitt lymphoma is a rare form of non-Hodgkin lymphoma associated with HIV infection, which classifies the patient in the AIDS stage. The rapid progression to dissemination or amelioration of the patient's condition depends on the promptness of initiating the HAART-associated with cytostatic therapy.

## Author contributions

**Conceptualization:** Victoria Birlutiu.

**Data curation:** Victoria Birlutiu, Ioan Sorin Zaharie, Mariana Sandu.

**Formal analysis:** Victoria Birlutiu, Rares Mircea Birlutiu, Ioan Sorin Zaharie, Mariana Sandu.

**Investigation:** Victoria Birlutiu, Rares Mircea Birlutiu, Ioan Sorin Zaharie, Mariana Sandu.

**Project administration:** Victoria Birlutiu.

**Resources:** Victoria Birlutiu.

**Validation:** Victoria Birlutiu, Rares Mircea Birlutiu.

**Visualization:** Victoria Birlutiu, Rares Mircea Birlutiu.

**Writing – original draft:** Victoria Birlutiu, Rares Mircea Birlutiu.

**Writing – review & editing:** Victoria Birlutiu, Rares Mircea Birlutiu.

## Supplementary Material

Supplemental Digital Content
