# Supplementary material for: Burkitt lymphoma associated with human immunodeficiency virus infection and pulmonary tuberculosis: A case report
Source: Medicine (Baltimore). 2020 Dec 24;99(52):e23853. doi: 10.1097/MD.0000000000023853 (PMC7769298; doi:10.1097/MD.0000000000023853)
Supplement: Supplemental Digital Content [file medi-99-e23853-s001.docx]

**Burkitt’s lymphoma associated with HIV infection and pulmonary tuberculosis: a case report.**

Victoria Birlutiu

Full CT scan re-assessment description:

A CT scan re-assessment of the patient was performed, which revealed the following changes: at cranial level, a right intraorbital tissue mass of 1/2cm, which imprinted and deflected the lateral rectus muscle of the right ocular globe with subtle modifications of adjacent bone structure and tissue component, intracranial, dural, mirror image, at the right temporal pole, with dimensions of 0.7/2.5 cm. Several nodular masses with development at the entire scalp level, some with the same changes in the adjacent bone structure and with an intracranial dural mass component, the largest at the right occipital level, with extracranial component of 1/4cm and the intracranial one, of 0.8/2,3cm. Changes in bone structure with millimetric cortical discontinuities in both mandibular condyles. The amorphous tissue component underlying the right temporal bone with bone changes at the level of the right temporal bone and right jugular foramen contours.

The native thoraco-abdomino-pelvic multiple detector computed tomography (CT) scan with contiguous sections revealed bilateral pleural effusion; tissue mass with the osteomuscular component developing at the tip of the left shoulder with lysis and the adjacent muscle swelling; similar masses at ribs V, VII, and IX on the left side. Right axillary adenopathies up to 3 cm. Paravertebral T10-11 tissue mass. Homogeneous hepatosplenomegaly. Bilateral adrenal nodes, 6 cm in diameter on the right, 4 cm on the left. Retroperitoneal paraaortic distal lymph node metastasis of 3.3 cm. Alterations of the left iliac muscle and left sacral wing. The entire posterior paravertebral lumbar muscle appeared with an amorphous structure. Osteolytic areas in the bilateral femoral neck and the transverse and spinous apophyses of the L4 vertebra, possibly with intrathecal evolution.
